# The Public Health

**Published:** 1918-09-21

**Authors:** 


### THE PUBLIC HEALTH.

### The Nursing of Measles.

Measles, when severe in type, may give rise to a relatively high case mortality in infants and young children. To combat this health authorities are now including in their Schemes of Maternity and Child Welfare arrangements for the nursing of measles. For obvious reasons health visitors and sick nurses cannot usually be employed for this purpose, and it is therefore desirable that special nurses should be appointed. In some districts Health Authorities have entered into agreements with Nursing Associations to provide nurses for the nursing of cases of measles, when and where required; in other districts special whole-time nurses are being appointed for the purpose. The great danger to infants and young children from an attack of measles is chiefly due to the pulmonary complications which are liable to develop; but this danger can be greatly minimised by early medical treatment and efficient nursing. Parents require to be instructed in the possible dangers which may accompany what by them is regarded as a simple attack of measles.

### Diagnosis of Malaria.

In the Report recently issued by the Local Government Board there is a comprehensive paper by Lieut.-Colonel S. P. James on the diagnosis, treatment, and prevention of malaria. The following are some of the more important points mentioned in the paper with reference to symptoms and diagnosis. Three clinical types of malarial fever are met with: (*a*) A comparatively mild type with regular paroxysms of fever every third day due to the benign tertian parasite. (*b*) A severe type with paroxysms of fever recurring every day and at irregular times, although theoretically they should recur every third day as in the first type. This type is caused by the malignant tertian parasite. (*c*) A comparatively mild type with regular paroxysms of fever recurring every fourth day; this type is produced by the quartian parasite. In the second or severe type occur pernicious symptoms which are referable to the alimentary system and abdominal organs, the circulatory system, the nervous system, and the urinary system. With regard to the causation of the febrile paroxysms it is believed that while the malarial parasite is growing inside the red-blood corpuscle and feeding on its hæmoglobin it elaborates a fever-producing chemical substance which as soon as the parasite bursts from the red cell escapes into the plasma and produces the febrile paroxysm. The diagnosis of malaria may be based on the following: (*a*) The clinical signs and symptoms, (*b*) the result of examination of the blood, and (*c*) the effect of therapeutic doses of quinine. With regard to the clinical signs the most important diagnostic indication is periodicity of a tertian or quartian type. The diagnosis of malaria may be made with certainty if fever or other symptoms recur at intervals of forty-eight or seventy-two hours. The microscopic examination of the blood provides a reliable method for arriving at an accurate diagnosis; it is necessary, however, that no quinine should be administered prior to the taking of the blood film. If other diagnostic methods have failed, the effect of therapeutic doses of quinine may be tried, although this should not be employed as a routine measure. If a case of fever resists 10 grains of quinine in solution thrice daily for two successive days it is most probably not a case of malarial fever.

### The Housing Problem.

The history of housing presents an interesting and instructive study. Up to within comparatively recent years the question of housing in this country was regarded almost entirely from the standpoint of domiciliary accommodation, and the relationship between housing conditions and national health was in no way sufficiently appreciated. The question, however, has now become a public health problem. The public mind has at last come to grasp the true significance of the relationship which exists between insanitary houses and the presence in our midst of certain forms of disease. For years the medical profession has been advocating widespread and drastic changes in the housing conditions of the industrial classes, but such advocacy has up to the present met with very inadequate response. Now, however, it is bearing fruit, and the truth is becoming more universally appreciated that if a high level of national health is to be attained a high standard of hygienic and sanitary home conditions must be secured.

### Half a Million Houses Required.

In consequence of the war the building of houses for the working classes has practically been in abeyance, and after peace is declared much new building will have to be undertaken. It is estimated that in England and Wales the erection of 500,000 houses will have immediately to be proceeded with. It is to be hoped that this important undertaking will not be commenced in any hurried, haphazard manner, and that when consideration is being given to the sites, structure, and arrangements of the new houses due regard will be paid to the health requirements of the people.

